# Deformation Behavior Investigation of Auxetic Structure Made of Poly(butylene adipate-co-terephthalate) Biopolymers Using Finite Element Method

**DOI:** 10.3390/polym15071792

**Published:** 2023-04-04

**Authors:** Yanling Schneider, Vinzenz Guski, Siegfried Schmauder, Javad Kadkhodapour, Jonas Hufert, Axel Grebhardt, Christian Bonten

**Affiliations:** 1 Institute for Materials Testing, Materials Science and Strength of Materials (IMWF), University of Stuttgart, Pfaffenwaldring 32, D-70569 Stuttgart, Germany; 2 Department of Mechanical Engineering, Shahid Rajaee Teacher Training University, Tehran P. O. Box 16785-163, Iran; 3 Institut für Kunststofftechnik, University of Stuttgart, Pfaffenwaldring 32, D-70569 Stuttgart, Germany

**Keywords:** auxetic behavior, metamaterial, biodegradable, Poisson’s ratio evolution, stress distribution, FE simulation

## Abstract

Auxetic structures made of biodegradable polymers are favorable for industrial and daily life applications. In this work, poly(butylene adipate-co-terephthalate) (PBAT) is chosen for the study of the deformation behavior of an inverse-honeycomb auxetic structure manufactured using the fused filament fabrication. The study focus is on auxetic behavior. One characteristic of polymer deformation prediction using finite element (FE) simulation is that no sounded FE model exists, due to the significantly different behavior of polymers under loading. The deformation behavior prediction of auxetic structures made of polymers poses more challenges, due to the coupled influences of material and topology on the overall behavior. Our work presents a general process to simulate auxetic structural deformation behavior for various polymers, such as PBAT, PLA (polylactic acid), and their blends. The current report emphasizes the first one. Limited by the state of the art, there is no unified regulation for calculating the Poisson’s ratio ν for auxetic structures. Here, three calculation ways of ν are presented based on measured data, one of which is found to be suitable to present the auxetic structural behavior. Still, the influence of the auxetic structural topology on the calculated Poisson’s ratio value is also discussed, and a suggestion is presented. The numerically predicted force–displacement curve, Poisson’s ratio evolution, and the deformed auxetic structural status match the testing results very well. Furthermore, FE simulation results can easily illustrate the stress distribution both statistically and local-topology particularized, which is very helpful in analyzing in-depth the auxetic behavior.

## 1. Introduction

Metamaterials are artificial structures and possess different permittivity and permeability from those in nature. Periodicity is one of their typical characteristics, since the metamaterials are usually made through repeating a single cell or element in the space. In addition, electrically or magnetically effective materials will be inserted inside the metamaterial. In addition to the material properties, the geometrical characteristics can also play an essential role in determining components’ overall load-bearing and deformation behavior. Auxetic structures, also called lattice structures, and materials possess a negative Poisson’s ratio, which means that the lateral expansion will appear under tension [1]. It means they exhibit counter-intuitive deformation characteristics. The Poisson’s ratio of an isotropic material is given as

(1) $$\nu =-\frac{\varepsilon_{yy}}{\varepsilon_{xx}}$$

with $\varepsilon_{xx}$, the tensile strain in loading direction and $\varepsilon_{yy}$ the tensile strain perpendicular to it ($\varepsilon_{xx}$) [1]. Wojciechowski [2] began with the generalized free energy description in elastic deformation by using the (Lagrange) strains in a D-dimensional isotropic medium. Then, the bulk modulus is formulated using Lamé coefficient λ, μ (shear modulus) and D:

(2) $$K=\lambda +\frac{2\mu }{D}.$$

In 3D [3], the Poisson’s ratio ν is

(3) $$\nu =\frac{DK-2\mu }{(D-1)DK+2\mu }=\frac{\lambda }{(D-1)\lambda +2\mu }.$$

Poisson’s ratio can be related to Young’s modulus E, bulk modulus K, and dimension D. For metamaterials, e.g., auxetic structures, no unified function to calculate the Poisson’s ratio exists. Ren et al. [4,5] presented a Poisson’s ratio function of a perforated sheet system based on the rectangle edge lengths (individual lattice structure) and the angle between the rectangles. Almgren [6] described the Poisson’s ratio for the 2D inverse honeycomb structure by using the hinge lengths, *a* and *b*, and their relaxed angle α. With the given conditions of equal strains in all axial directions and no shear strain (shear modulus infinity), Almgren [6] deduced the Young’s modulus dependent on *a* (a=b) and α in 2D and 3D. Ai and Gao [7] studied the Poisson’s ratio behavior for metal metamaterials by using the whole structural data (not based on individual lattice structure). Pandini and Pegoretti [8] presented equations to calculate the Poisson’s ratio and its rate for the tensile specimens made of poly(butylene terephthalate). Basically, the strains in transverse and loading directions are used. They found that Poisson’s ratio displays the typical features of a retardation function. For idealized 2D cellular materials, Gibson et al. [9] derived the linear-elastic moduli and elastic and plastic collapse stresses. Such properties can be related to the cell-wall properties, cell shape, and density. It also presented the Poisson’s ratio using the cellular geometrical parameters [9]. The auxetic structures that appeared most were 2D or 3D. Still, most of such structures are designed for macro models. It also means some designed auxetic structures are desired for the microscale. Concerning the category of auxetic structures, the review article in Wang et al. [10] listed seven groups, the first six of which included various unit cell types, and the last comprised other types that have appeared in the literature. The first six groups are re-entrant, chiral, rotating, origami-based and kirigami-based, perforation, and foam structures. A traditional unit cell of the re-entrant type is an inverse honeycomb cell. Others are double-arrowhead, three-star-shaped (also four-, six-, i.e., N-star-shaped), lozenge grid, etc. Some examples of chiral structures include trichiral, tetrachiral, antitetrachiral, and hexachiral. For more examples and detailed information about the auxetic structures of the six groups mentioned above, refer to Wang et al. [10]. The seventh group lists some other novel auxetic structures, e.g., interlocking hexagonal model, 3D soft auxetic metamaterials (“bucklicrystals”), and micro- and nano auxetic metamaterials such as α-cristobalite SiO${}_{2}$. Generally, and more or less, various investigations have been performed for each appeared unit cell, e.g., [11,12,13,14,15].

Only a few natural cellular auxetic structures/materials have been found until now, such as silicon dioxide (SiO${}_{2}$), the peel of the pomelo [16,17], and silk fibers [18]. Most existing auxetic structures are so-called mechanical metamaterials [15]. Lakes [19] was among the first to report an effective method to fabricate re-entrant foams with a negative Poisson’s ratio of around −0.7 in the year 1987. Here, “re-entrant’’ means the angles formed by the diagonal struts in the unit cell point inwards [15]. Since Lakes [19], many other types of auxetic metamaterials (structures) have been reported by researchers, such as chiral and rotational rigid structures [4]. Cellular auxetic metamaterials are composed of repeated unit cells. Auxetic metamaterials have many preferable properties, such as high indentation, shear, fracture resistance, and good energy absorption capacity. Generally, such structures show relatively low stiffness. Kelkar et al. and Negrea [20,21] present a review of auxetic metamaterials (structures). The potential applications of metamaterials can be in civil engineering, protective engineering, medical treatment, and intelligent materials. Conventional plastics, one of the essential materials, are widely used in various fields, owing to their excellent material properties, with respect to lightweight and low costs. However, such materials, e.g., polyethylene and polystyrene, originate from petroleum, and their degradation is also a difficult issue [22]. The production of conventional plastics in large amounts causes high consumption of nonrenewable resources and environmental pollution. On the other hand, this global plastic waste problem positively influences the development of biodegradable biopolymers and other sustainable materials [23]. Poly(butylene adipate-co-terephthalate) (PBAT) is a kind of 100% biodegradable polymers. Polymers can be reinforced with fibers or particles. Polylactic acid (PLA) is another kind of 100% biodegradable polymer with a much higher strength than PBAT. Agaliotis et al. [24] reported a new kind of natural fiber-reinforced composite (NFRC) filament fabricated using PLA reinforced with flour of henequen (a kind of plant) fibers. They studied the effect of the flour content on the tensile properties, including thermal, physical, and microscopic characteristics, where the specimen is manufactured by 3D printing [24]. Additive manufacturing (3D printing) techniques are currently used to produce auxetic structures. These techniques allow for the manufacturing of components by adding materials layer-by-layer from a CAD design [25]. Such a process enables the integration of design and manufacturing processes, efficient use of materials with minimal waste, and easy production of highly complex geometries [26]. A review of 3D printing, including methods and product property characteristics, can be found in [27,28,29]. Lee et al. [30] presented a review of the NFRC produced by FDM. Mainly, it aimed to promote the study or usage of kenaf fiber reinforcement in PLA composite filaments.

In the field of material behavior investigation, numerical simulation can play a role as important as the experiment. For the deformation behavior prediction of plastic materials or structures, Bergström and Boyce [31] advanced a successful approach (theory) based on continuum mechanics to model the elastomeric rate-dependent material behavior. Their further work [32] extended this constitutive model to account for the effect of filler particles, such as carbon black, on the time-dependent, hysteretic stress–strain behavior. Some researchers attempted to use J${}_{2}$-plasticity to simulate the behavior of ultra-high molecular weight polyethylene (UHMWPE) [33], an important thermoplastic. However, this model does not accurately capture the complex set of experimental behaviors of UHMWPE. Bergström and Bischoff [34] developed and validated a new and more accurate and computationally efficient thermomechanical material model for thermoplastic materials, particularly UHMWPE. Garzon-Hernandez et al. [25] presented an approach that aims at complementing the 3D printing process with a continuum model to describe the macroscopic behavior of fused filament fabrication (FFF) thermoplastics, while preserving links with printing parameters. FFF is a 3D printing method, also called fused deposition modeling (FDM). Their model was formulated in finite deformations within a thermodynamically consistent framework. Mirkhalaf and Fagerström adopted an elasto-viscoplastic constitutive model [35,36,37,38], developed in a finite strain setting, to model the mechanical behavior of PLA. Dal and Kaliske [39] proposed an algorithmic setting for Bergström-Boyce [31] finite viscoelasticity model suitable for the FE simulation. Their work [39] also revisited the thermodynamical requirements and proved the consistency of the model based on dissipation inequality.

This work experimentally and numerically studies the deformation characteristics of auxetic structures made of biodegradable and environment-friendly PBAT. The inverse honeycomb shape, a simple case of the re-entrant structure, is preferred. The currently used metamaterial is commercially available. For a detailed description of the experiments, refer to Hufert et al. [40]. The finite element (FE) calculations are performed on tensile and auxetic specimens. In order to find a suitable inherent model in ABAQUS, the measured tensile stress–strain flow curve of a standardized tensile specimen (DIN EN ISO 527-1) is used to calibrate the FE result. Consequently, the found model would be used to predict the deformation behavior of 2D auxetic structure. The comparison between experimental and numerical results includes the deformed structural status, the Poisson’s ratio evolution, and the force–displacement curve. This work contributes to finding suitable FE models to numerically simulate the deformation behavior of auxetic structures made of various polymers, since no generally applicable FE model(s) exist(s), due to the limitation of the state of the art. Such found FE models are inherent functions in software (here ABAQUS), the application of which is easy for all researchers and engineers. The simulation difficulty comes from two aspects: auxetic behavior not fully understood yet (topological/structural aspect) and the significantly different behavior of polymers under loading (material aspect). The latter embodies lack of a sophisticated FE model (theory) to describe various polymer deformation behavior. A suitable FE model is found if the simulated global stress–strain flow behavior matches the measured data well (a tensile specimen). Consequently, the auxetic structural behavior prediction will use the same FE material model. This process is successfully applied for PBAT, PLA, and their blends. Here, the results for PBAT will be shown, and the others will be presented in subsequent works. Another contribution is that three calculation ways of Poisson’s ratio ν are presented based on measured data, one of which is found to be suitable to present the auxetic structural behavior. Still, the FE-predicted Poisson’s ratio evolution calculated using the same method matches the experimental data very well. Furthermore, both experiment and simulation found that at least 5 × 5 cells are required to describe the representative value.

## 2. Materials and Experiments

### 2.1. Materials

Our study aims to find plastic compounds with preferable properties, such as good ductility, high strength, and being environment friendly. The basic materials and components of compounds should be commercially available. The final goal is to produce auxetic structures with desired performances. For such investigations, various tests and simulations are executed. PLA and PBAT polymers are chosen since they are 100% biodegradable and are mutually good blending partners for each other. Still, PLA shows relatively high strength and low ductility, while PBAT does the other way around. The blend of both polymers allows for the modification of the mechanical properties, such as the stiffness and maximum elongation. PLA and PBAT belong to thermoplastics among elastomer, duroplastic, and thermoplastic. The current work focuses on the PBAT deformation properties/behaviors achieved from experiments [40] and FE simulations. Here, only a short introduction is given. Commercial PBAT Ecoflex F Blend C1200, a fully biodegradable plastic, was purchased from BASF, Ludwigshafen, Germany. A schematic illustration of the PBAT’s chemical structure can be referred to [22,41].

The filaments are produced from pellets by the authors. The compounding was carried out on an EBVP25 twin screw extruder (O.M.C., Saronno, Italy). The screw design of the compounding process included different kneading and mixing elements [40]. A 30 × 25D single-screw extruder (COLLIN Lab and Pilot Solutions GmbH, Maitenbeth, Germany) was used for filament extrusion. The filaments were extruded with a diameter of 1.75 mm at a speed of 25 1/min. The filament diameter was continuously determined and checked with an ODAC 15XY laser measuring device (Zumbach Electronic AG, Orpund, Switzerland) [40].

### 2.2. Tensile Test

#### 2.2.1. Standard Tensile Test Specimen

**Figure 1:**
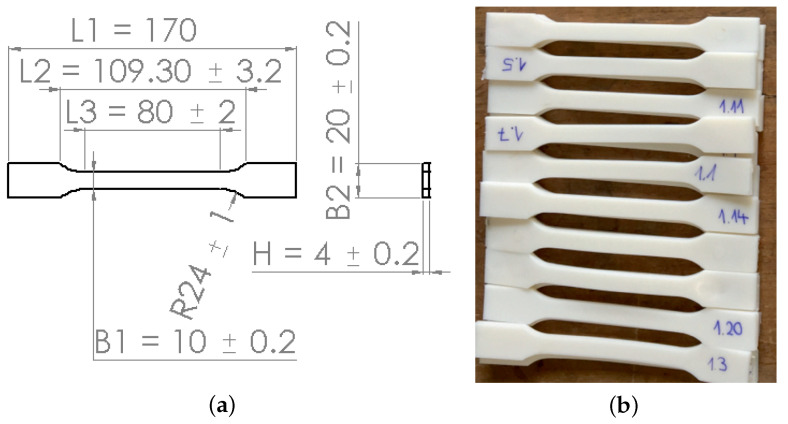
Geometry of the dog-bone-shaped tensile specimen according to DIN EN ISO 527-1 (unit: mm): (**a**) a sketch; (**b**) some printed specimens using injection molding method.

**Figure 2:**
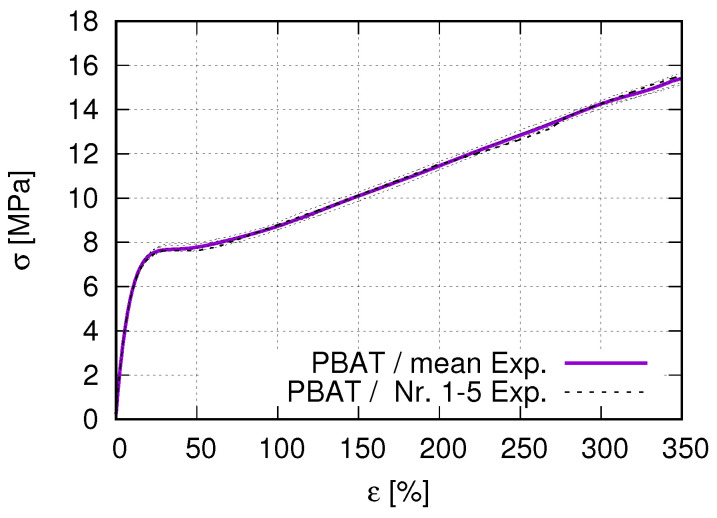
Tensile true stress–strain curves obtained by using the specimen geometry shown in Figure 1 for pure PBAT.

To determine the mechanical properties, tensile tests were carried out on a universal testing machine 1455 (ZwickRoell, Ulm, Germany). The test speed was set to 5 mm/min. for all tested specimens with a clamping length of 115 mm. For this purpose, tensile test specimens were produced using injection molding and 3D-printing methods, resulting in very similar material properties, such as the stress–strain behavior and Young’s modulus. Since the FE simulation uses the data from the former one, this work will show used technical parameters and measured results from the injection molding method. Table 1 lists the parameters used in the injection molding process. The tensile behavior of neat PLA and PBAT can serve as the comparative basis for their blends’ behavior. This study uses the tensile flow behavior to calibrate a suitable material model (theory) for successfully simulating the auxetic behavior. The geometry of the tensile test specimen is printed according to DIN EN ISO 527-1 standard. Figure 1a,b illustrates the geometry of the used dog-bone-shaped tensile flat specimen and some specimens produced by the injection molding process, respectively. All the performed tensile tests are strain-controlled in the current work. Figure 2 denotes the measured true stress–strain curve of the PBAT specimen shown in Figure 1. The test is performed five times. The purple solid line in Figure 2b is the mean value from all five stress–strain curves. The measured Young’s modulus is about 38.13 MPa and the corresponding yield stress $R_{p_{0.2}}$ = 7.76 MPa. Practically, the material yields before the 7.76 MPa stress is reached (Figure 2). After the yielding, a stress plateau zone appears for a strain range of approximately 25%, namely at about 25–50% loading strain. Beginning at about 50% strain, the stress increases again nearly linearly. There is no break at 350% strain, the maximum strain shown in Figure 2. The PBAT specimen shows no failure, even at 500% strain, the maximum load of the testing device. According to Ferreira et al. [42], PBAT with a Young’s modulus of 20–35 MPa and a tensile strength of 32–36 MPa possesses an elongation at the break of about 700% strain, which is higher than most other biodegradable polymers. Jiang et al. [43] reported a strain of 710% at failure for PBAT.

#### 2.2.2. Auxetic Tensile Test Specimen

**Figure 3:**
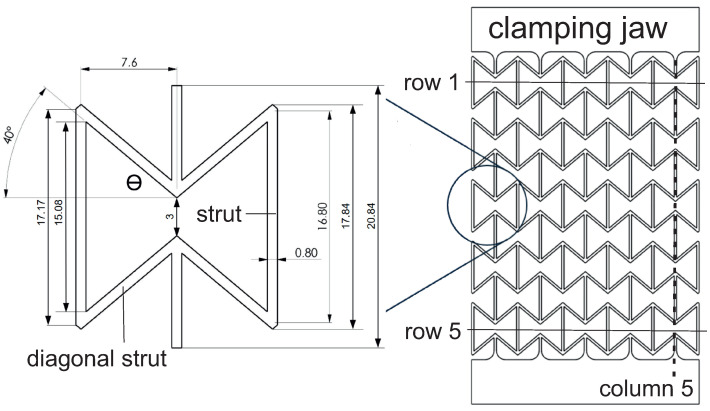
Schematic representation of a single honeycomb and its dimensions in [mm] in an auxetic structure with 5 × 5 cells. Figure reprinted from Ref. [40].

The deformation behavior of an auxetic structure depends on various factors, e.g., the material, the total number of unit cells, the type of the unit cell, and its detailed geometric parameters. The current work chose the inverse honeycomb shape, a type of re-entrant cells, as the unit cell to construct the auxetic structure (Figure 3). The specimens are manufactured by FDM. Table 2 lists some test data used in the printing process for the auxetic specimens.

**Figure 4:**
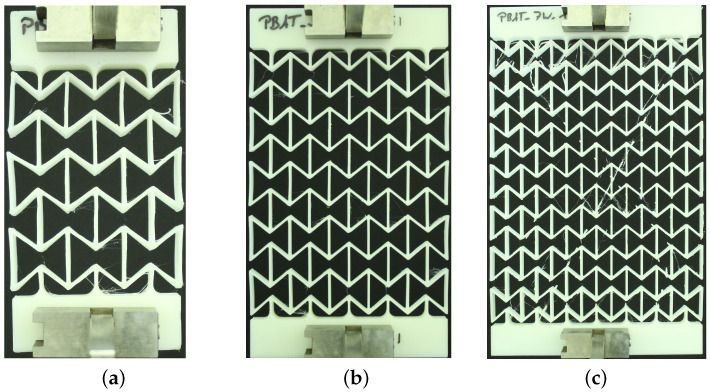
Printed auxetic structures made of PBAT mounted in the tensile loading facility: (**a**–**c**) with 3 × 3, 5 × 5, and 7 × 7 cells in a scaled view at slightly loaded status (loading strain ≤ 3%), respectively.

**Figure 5:**
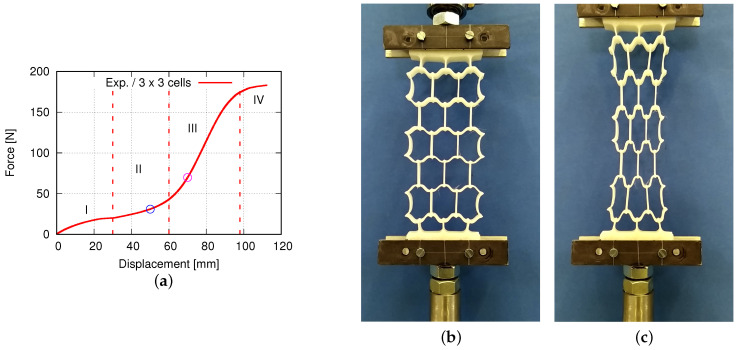
(**a**) Tensile force–displacement curve of PBAT auxetic structures composed of 3 × 3 cells with the blue and magenta circles corresponding to (**b**,**c**), respectively; (**b**,**c**) deformed status with 50 mm and 70 mm loading displacement, respectively. (RIF, Dortmund/Germany).

Figure 3 displays the dimensions of the unit cell and a sketch of an auxetic structure with 5 × 5 cells used in this study. Such a specimen is called 2D, since the third dimension in Z direction is extruded with the 2D geometry in the X-Y plane. The strain-controlled tensile tests performed on auxetic structures are executed on specimens with different total numbers of cells, namely 1 × 1, 3 × 3, 5 × 5, and 7 × 7, and no less than five times for each case. Figure 4 demonstrates three printed auxetic structures with 3 × 3, 5 × 5, and 7 × 7 cells, which are mounted in the tensile loading machine at slightly loaded status (loading strain ≤3%). Figure 5a denotes the force–displacement curve for a structure with 3 × 3 cells. The curve evolution presents four regions, I–IV, as marked with red dashed lines (Figure 5a). The first region I covers about the loading region of 0 mm to 30 mm, where the force increases corresponding to the loading increment, and the curve is convex. The second region II is from 30 mm to 60 mm. As in region I, the force increases at a moderate rate in region II, but the force–displacement curve is concave. In regions I and II, it also shows a plateau from about 25 mm to 35 mm. These two regions could be auxetic deformation related. Compared to region I and II, region III illustrates a rapid force increment. The increment is about 42 N by 60 mm loading for the former, while 132 N by further 38 mm loading for the latter. After this rapid force increment in region III, the curve illustrates another plateau, a slow force-increasing rate. The specimen is not broken at the given max loading in Figure 5a. Figure 5b,c present the deformed status of the specimen at 50 mm and 70 mm loading, which correspond to the blue and magenta circles in Figure 5a. The structural status in Figure 5b nearly reaches the auxetic deformation limit, i.e., the Poisson’s ratio of the structure is still negative. Figure 5c is not auxetic behavior relevant anymore, but proves the extraordinary ductility of PBAT.

**Figure 6:**
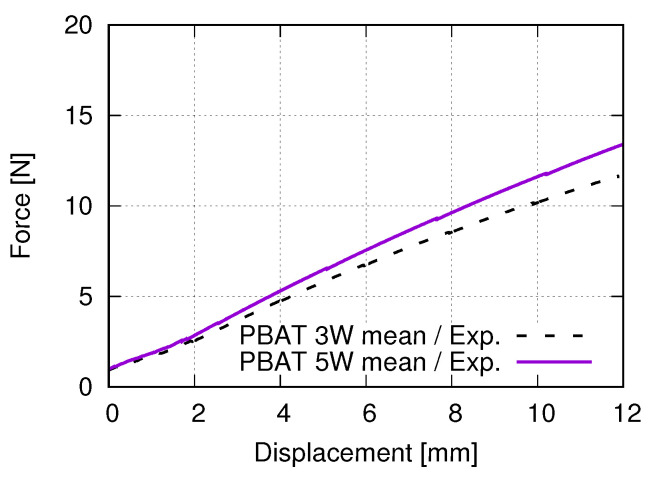
Force–displacement curves of PBAT auxetic structures composed of 3 × 3 and 5 × 5 cells, force of which is the mean value from three tests under strain controlled tensile tests.

One objective of this work is to find out the optimum cell number in an auxetic structure to represent the actual auxetic behavior for the laboratory investigation, since the total number of unit cells affects the structural deformation behavior and can lead to deviating results. The optimum one would be selected from 3 × 3, 5 × 5, and 7 × 7 cells. For the case of only one or four (2 × 2) cell(s) in the structure, there is no cell free of boundary conditions (BCs). BCs introduce abnormal deformation, especially for the cells as neighbors of boundaries. For structures with more than 49 (7 × 7) cells, the required material and printing time would be large, i.e., neither economical nor time efficient. The experimental results showed that a structure with 5 × 5 cells is preferable [40], since its deformation behavior is similar to the one from 7 × 7 cells, while 3 × 3 could not accurately catch the deformation behavior. On the other hand, it needs only about one-half of the materials, compared to 7 × 7 cells, without considering the clamping jaws. Due to the very small cross-section area of such auxetic structures (Figure 3), the calculated stress would be very high, which is misleading for the strength of such structures. In this work, the force–displacement curve presents the tensile flow behavior. It is observed that tiny deviations of the force/stress value at a given displacement exist among repeated tests. Generally, such small deviation is negligible, compared to the measured total value. Figure 6 illustrates the tensile force–displacement curves of structures with 3 × 3 (“3W”) and 5 × 5 (“5W”) cells. The data for each curve are mean values from three tests. It is pointed out that Figure 6 only shows the measured result partly. Until about 2 mm displacement, the applied forces are nearly identical for both curves and very similar between 2 to 4 mm displacement. After that, and at about 12 mm displacement, the structure with 5 × 5 cells needs more force than the one with 3 × 3, but the largest difference is only about 1.7 N, with 3 × 3 cells approximately 11.54 N and 5 × 5 cells 13.24 N. The slightly higher force, $R_{force}=\frac{13.24}{11.54}\approx 114.73\%$, is non-proportional to the ratio calculated according to the number of total cells (mass) $R_{cell}=\frac{25}{9}\approx 277.78\%$ and much lower. It is expected that the applied force $F_{25cells}$ of the structure with 5 × 5 cells would be lower than the value 277.78%F${}_{9cells}$, due to the influence of structural geometry, but not as low as about $114.73\%F_{9cells}$. Further study is necessary to explain the force-cell ratio behavior quantitatively.

#### 2.2.3. Poisson’s Ratio Calculation from Measured Data

**Figure 7:**
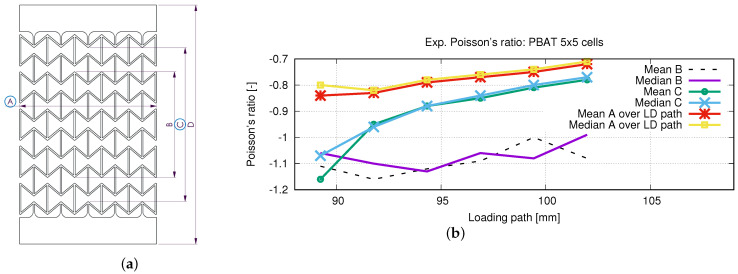
(**a**) A sketch to present the used lengths to calculate the Poissobn’s ratio by using three different methods; (**b**) according to loading, experimental Poisson’s ratio evolution calculated by three different methods.

One of the essential characteristics of the auxetic structural deformation behavior is the Poisson’s ratio. To the authors’ knowledge, no international standard method is available for calculating the Poisson’s ratio of the auxetic structure. In this work, three different approaches are used. Figure 7a shows the necessary lengths used for the calculation, and Figure 7b plots the results. A, B, C, and D in Figure 7a present the width of the whole structure, the length of the middle three rows, the specific length of five rows, and the total specimen length. For lengths indicated by A to D, the alteration would be firstly calculated for each concerned row from which the engineering (nominal) strains are calculated. The Poisson’s ratio is obtained by applying Equation (1). In this case, “A” is used in calculating $\varepsilon_{xx}$ and B to D $\varepsilon_{yy}$. All the calculated evolutions of the Poisson’s ratio illustrate an increasing tendency according to the loading. In Figure 7b, “mean” presents that the plotting value is taken from the mean value among all the measured data, while “median” presents the median value of the measured minimum and maximum values. The two curves in Figure 7b, marked with “Mean B” and “Median B”, behave zig-zag-like. Still, the calculated Poisson’s ratios are mostly smaller than −1, which implies that the lateral deformation (strain) is more significant than the one in the loading direction. The curves marked with “Mean C” and “Median C” show Poisson’s ratio with values mostly greater than −1. The last two curves present few differences, and the one with mean value is preferred to present the Poisson’s ratios of the auxetic structure, where the “LD path” presents the loaded displacement of the whole specimen. Observed from both tests and simulations, the deformation of the two rows, as neighbors of clamping jaws, is influenced by the BCs too much. It causes a large deformation discrepancy, compared to the inside rows, which is also a reason why rows 1 and 5 (Figure 3) are excluded in Poisson’s ratio calculation for the auxetic behavior.

## 3. FE Simulation

As mentioned above, the current work aims to experimentally and numerically investigate the auxetic deformation behavior. For the latter, it is preferred to use the FE simulation. For an in-depth study, the initial structure can be reconstructed from the tomographic images (real structure), including initial damages, such as voids, holes, and cracks. Still, the residual stresses introduced by the printing process and cooling down is considered. An ideal material model (theory) would be able to describe the deformation behavior of all considered polymers, i.e., PLA, PBAT, and their blends in the current case. Due to limited time, testing facility, and the state of the art of theories describing polymers’ deformation behavior, the current work will use the geometry from CAD design as the initial structural status and load the auxetic structure, without taking residual stress into account. The residual stress and the real auxetic structure will be introduced to the FE simulations in the consecutive reports.

Still further work is necessary to achieve a well-proved theory suitable for describing the deformation behavior of polymers in the same category mentioned above (PLA and PBAT belonging to thermoplastic). The current study uses ABAQUS [44,45] as a solver for the FE simulation. The inherent material models in ABAQUS will be selected. A suitable one would be found if the tensile stress–strain curve of the FE result matches the experimental one (Figure 2) well. It means that this material model will be applied in the FE simulation to predict the auxetic structural deformation behavior. In our trial simulations, a single material model (ABAQUS) is not found that can accurately describe the deformation behavior of PLA, PBAT, and their blends. Three different material models are applied, all of which are theoretically suitable for describing the plastic/polymer/rubber deformation behavior [45]. As mentioned above, this work concentrates on the deformation behavior of auxetic structures made of PBAT. A part of the simulation results, including the parameter identification, is from the master theses [46,47], where both works were done under the current authors’ supervision.

### 3.1. Material Model

Several ABAQUS-inherent material models, such as “$J_{2}$”, “Marlow”, “Yeoh”, “Ogden”, “Polynomial”, and “Reduced Polynomial” [48,49,50], are applied in FE simulations using the geometry shown in Figure 1. It is pointed out that only the effective part is considered in the FE simulation, i.e., the clamping jaw and the notch excluded. Summarized instruction for some detailed descriptions of these models can be found by referring to [44,51]. For PBAT, the “Ogden” with N = 3, N = 4 [50] and “Marlow” [48] models resulted in a high agreement with the experimental one using an inverse engineering approach. Finally, the “Ogden” with N = 4 is chosen.

**Figure 8:**
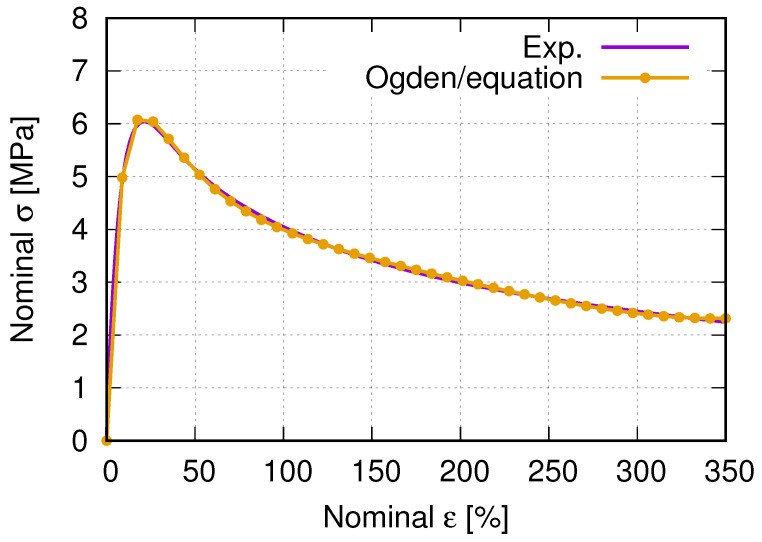
Nominal stress–strain curves of PBAT from the experiment and calculated by ABAQUS (Ogden, N = 4), parameters of which are given in Table 3.

The above-mentioned Ogden model is a particular form of the strain energy potential for the hyperelastic material model. In addition to Ogden, polynomial, Arruda-Boyce, and Van der Waals forms are available in ABAQUS as the incompressible or almost incompressible models. The current case belongs to the latter. The hyperelastic material behavior describes the deformation behavior of polymers and other rubber-like materials in ABAQUS. For such hyperelastic materials, the constitutive behavior is defined as total stress–total strain relationship different from that of the rate formulation in the context of history-dependent materials. In the current case, the total volume change of a given point *J* is defined as the determinant of the deformation gradient F, where *J* and F lead to $\bar{F}$ as the deformation gradient with the volume change eliminated. Furthermore, the deviatoric stretch tensor (the left Cauchy–Green strain tensor) can be defined by $\bar{F}$, where the first ($I_{1}$) and second ($I_{2}$) strain invariant can be defined. The strain energy potential defines the Cauchy (true) stress σ. From the virtual work principle, the internal energy variation is a function of *J*, σ, virtual rate of deformation (*D*), the current volume *V*, and the reference volume $V_{0}$. The strain energy *U* is a function of strain invariant ($I_{1}$ and $I_{2}$) and *J* for isotropic and compressible materials. The variation of the strain energy potential is the internal virtual work per reference volume. Since hyperelastic materials are often incompressible or nearly so, ABAQUS uses mixed (“hybrid”) formulations. It refers to ABAQUS [51] for more detailed description of the hyperelstic material behavior. Table 3 lists the used parameters for the FE simulation. The material contants $\mu_{i}$, $\alpha_{i}$ and $D_{i}$ are automatically calculated by ABAQUS-CAE using “uniaxial test data (nominal stress and strain)” and “isotropic material” methods. It means ABAQUS can calibrate these parameters if the test data are provided. Figure 8 illustrates the nominal stress–strain curves of PBAT from the experiment and calculated by equation (Ogden, N = 4), where the parameter of the equation is shown in Table 3. The exact formulation of the equation mentioned above refers to ABAQUS [44].

**Figure 9:**
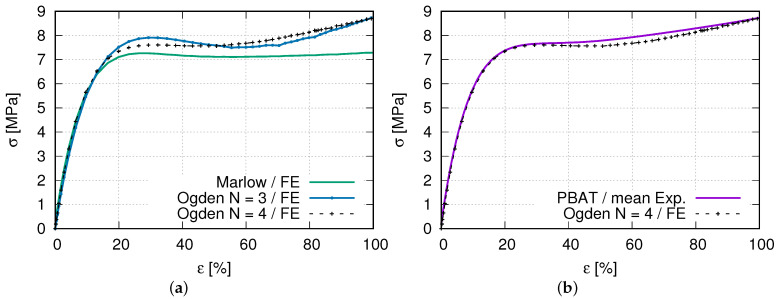
(**a**) FE simulated stress–strain curves for the selection of a best suitable material model (Ogden, N = 4) to describe PBAT deformation behavior; (**b**) comparison of experimental and FE stress–strain curves (Ogden N = 4).

Figure 9 illustrates the true stress–strain curves from FE predictions and the experiments. The model “Ogden N = 4” is selected to be applied in the FE simulation to predict the auxetic structure’s deformation, since it matches the testing curve best. Hosseini et al. [52] presented a review of the constitutive models for rubber-like materials.

### 3.2. Applied Structure and Boundary Conditions

**Figure 10:**
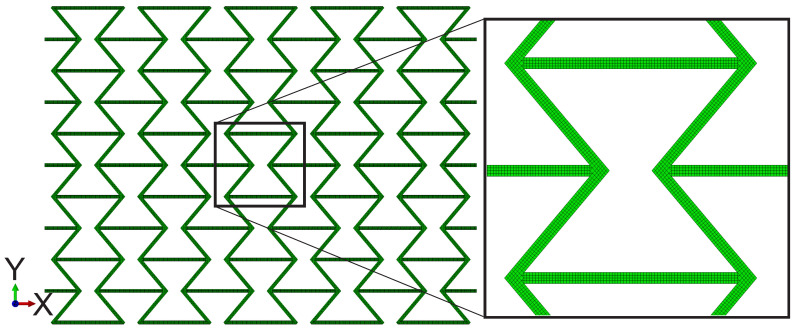
Meshing of the auxetic structures with 5 × 5 cells and a zoomed-in view.

The current goal is to study the auxetic deformation behavior; therefore, the testing and numerical results of the dog-bone tensile test specimens will not be discussed in the following section. The applied structure in the FE simulation is the auxetic structure with 5 × 5 cells and without the clamping jaw, a sketch of which is shown in Figure 3. Both element types of C3D8 and C3D8H are applied during trial calculations. It is found that the C3D8H type is more suitable for simulating the polymer behavior. Garzon-Hernandez et al. [25] proposed a hyperelastic constitutive model to predict the mechanical behavior of FDM thermoplastics. Figure 10 illustrates the whole structure’s meshing, where the center cell’s meshing is presented in a zoom-in view. There is a total of 738,760 octagonal and hexagonal elements with types of C3D8H and C3D6H. The edge size of most C3D8H elements is 0.2 mm in all three directions. In the wending position of two struts, a slightly larger or smaller edge size than 0.2 mm exists. The thickness in the third direction is 5 mm, i.e., 25 layers. It also implies that half of the specimen thickness is applied in the simulation. On the one hand, it is limited by the ABAQUS calculation capacity. Based on experience, ABAQUS can only correctly start the calculation with less than one million elements. On the other hand, the half-thickness is very time efficient, with negligible influence on the FE result accuracy. The homogeneous boundary conditions (BCs) are assigned according to the testing conditions applied on the specimen.

### 3.3. Poisson’s Ratio Calculation from FE Results

**Figure 11:**
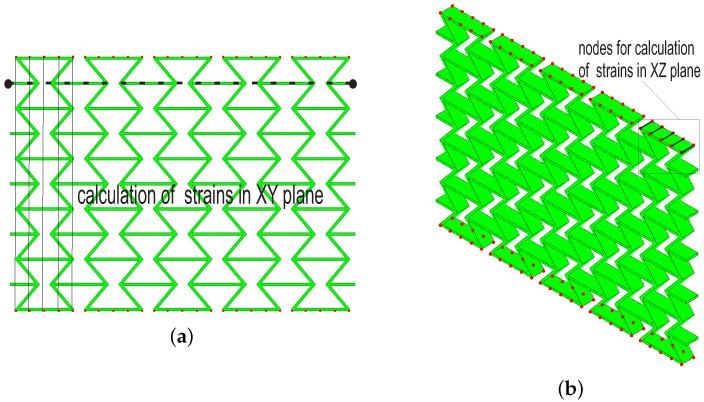
Calculation of Poisson’s ratio from FE results: (**a**) node pairs to calculate strains in XY plane; (**b**) analogous to (**a**), but for XZ plane.

As given in Section 2.2.3, Equation (1) is used for Poisson’s ratio calculation of FE results. Figure 11 shows the lengths necessary for the Poisson’s ratio calculation, where results of Figure 11a are comparable with experimental ones ($\nu =-\frac{\varepsilon_{yy}}{\varepsilon_{xx}}$). Figure 11b is only to show that Poisson’s ratio in the XZ plane can also be deduced ($\nu =-\frac{\varepsilon_{zz}}{\varepsilon_{xx}}$), which is not used in this work. The horizontal direction (XX) is the loading direction, where it refers to Figure 10 for the coordinates. Only one strain value in the XX direction is necessary, since the strains are all the same, due to homogenous boundary conditions. Five pairs of nodes in each row are selected in the YY direction, and the strains in the lateral direction are deduced. At each deformed status, the mean value of the calculated strains (XX) is used for the structural Poisson’s ratio deduction. Shortly, the calculation of Poisson’s ratio for FE results is the same as the one used for measured data.

## 4. Measured and Simulated Results

**Figure 12:**
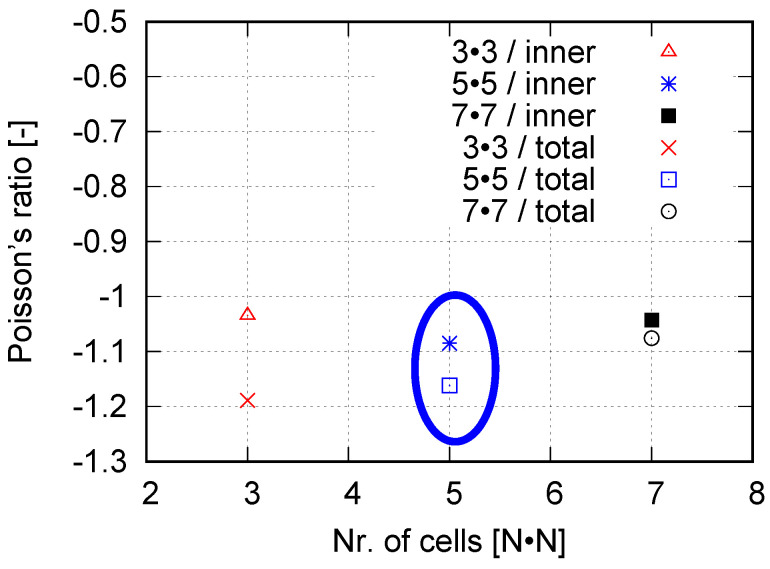
FE numerical proof of the 5 × 5 cells as the optimum one among auxetic structures with 3 × 3, 5 × 5, and 7 × 7 cells, taking ABS as the specimen material to show its independence of materials.

**Figure 13:**
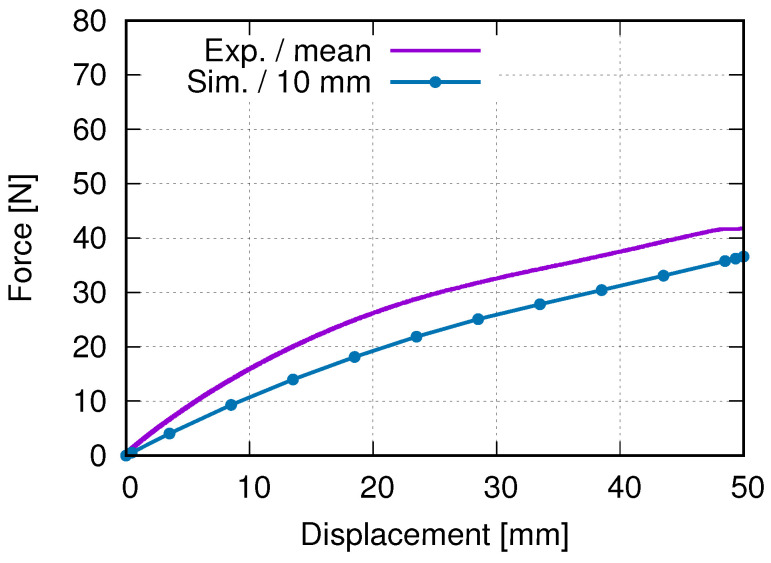
Experimental and FE-predicted force–displacement curves of PBAT auxetic structure with 5 × 5 cells.

**Figure 14:**
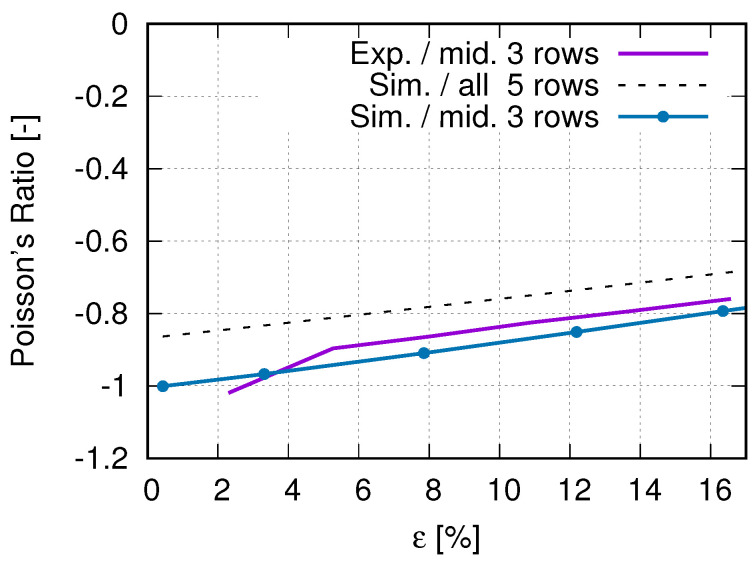
Experimentally measured and numerically predicted Poisson’s ratio development, according to loading for a PBAT auxetic structure with 5 × 5 cells. Experimental curve is reprinted from Ref. [40].

In order to obtain the optimized total cell number in an auxetic structure, 3 × 3, 5 × 5, and 7 × 7 cells are selected. Figure 12 presents their structural Poisson’s ratios for acrylonitrile butadiene styrene (ABS) [53,54]. It is pointed out that results shown in Figure 12 are from an early work, where the J${}_{2}$ theory was applied. It implies that the result shown in Figure 12 is material independent. This FE simulation (Figure 12) considered the residual stress, due to cooling down, which was predicted by the software Digimat-AM [55]. It is assumed that the Poisson’s ratio near −1 corresponds to the optimal structure in the sense of the lateral deformation. Even though the auxetic structure with 7 × 7 cells shows slightly better structural deformation behavior (Poisson’s ratio nearer to −1) than with 5 × 5 cells, the latter (5 × 5 cells) is taken as the better one. The reason is that the former (7 × 7 cells) needs a nearly double amount of materials and, thus, more costs. Based on further study results, other material models (than J${}_{2}$ theory) are applied to simulate the deformation behavior of PLA and PBAT polymers. As mentioned, the force–displacement curve is preferred to present the macro tensile deformation behavior of auxetic structures. Figure 13 illustrates the comparison of the force–displacement curves between the test and the simulation, where the FE-predicted forces needs a factor of two to be comparable with the experimental data. The measured curves from the experiment show a negligible small difference. Hence, the mean value of all the testing data are taken here. Figure 14 displays the experimentally measured and numerically predicted Poisson’s ratio evolution of the auxetic structure (Figure 3), according to the loading strain. The curve obtained from the average of five rows shows that the two rows as neighbors of the clamping jaw should be excluded in calculating the structural Poisson’s ratio, since the BCs influence their deformation too much. This effect of BCs leads to unusual auxetic behavior apparently. In Figure 14, only the numerical curve calculated from three rows is comparable to the experimental one.

**Figure 15:**
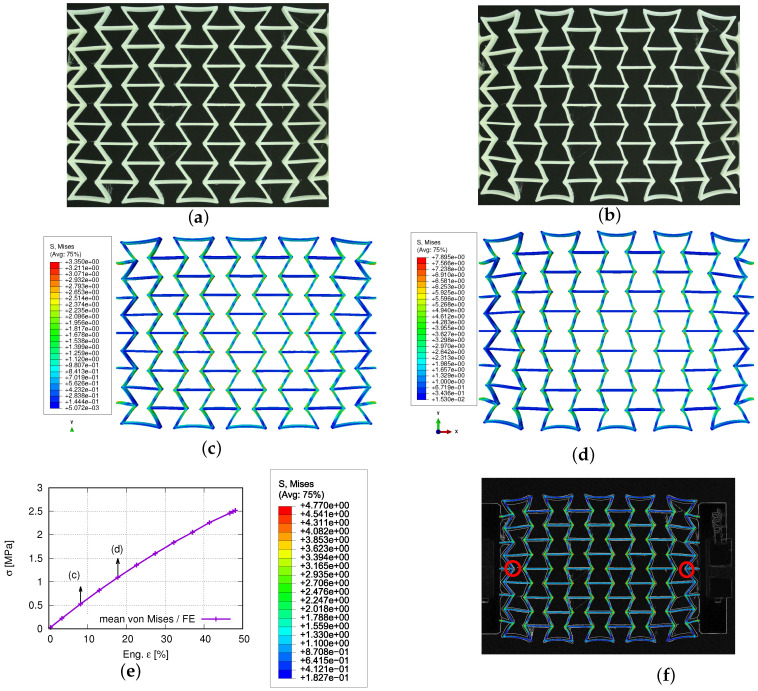
Experimentally measured and numerically predicted deformed status, according to loading for a PBAT auxetic structure with 5 × 5 cells: (**a**,**b**) measured at 8.8% and 17.6% strain, respectively; (**c**,**d**) FE-predicted von Mises stress at 8.2% and 17.8% strain, respectively; (**e**) FE-predicted mean stress evolution, according to loading; (**f**) comparison of deformed auxetic structure by overlapping experimental (gray contour) and FE results (colored area), where the legend only presents the FE result for the von Mises stress distribution.

Figure 15 compares some deformed status of the auxetic structure between the measured and FE-predicted results. Figure 15a,b are recorded at 8.8% and 17.6% loaded (engineering) strain from the experiment. Comparatively, the simulated ones are shown in Figure 15c,d, presenting 8.2% and 17.8% loading strain status for von Mises stress. Figure 15e plots the mean value evolution of the von Mises stress for the whole structure, according to the loading (engineering) strains. Figure 15f demonstrates an overlayed view, where the gray contour and colored area present the testing and predicted deformation, respectively. The legend in Figure 15f is valid only for the numerical result, which is the von Mises stress. The two highlighted nodes and the position of clamping jaws at both ends of the specimen prove the equal loading status in the test and the simulation. The distance between these two nodes is 99.8 mm in the simulation and 100.8 mm in the experiment.

**Figure 16:**
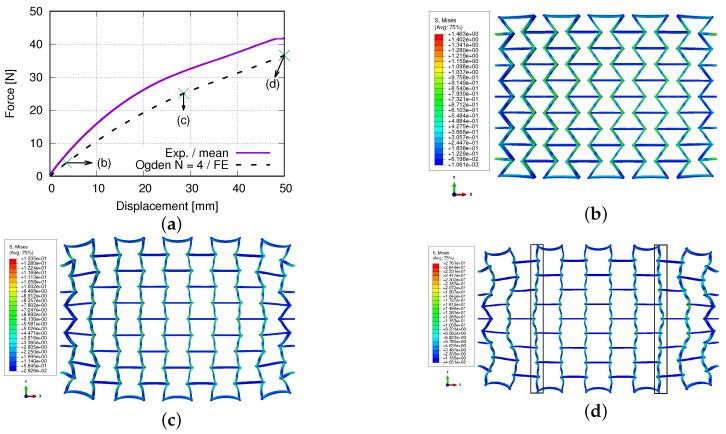
FE predicted von Mises stress and auxetic structure development, according to loading: (**a**) mean value evolution of von Mises stress, according to the (engineering) loading strain; (**b**–**d**) the deformed status of the auxetic structure at 3.37%, 27.42%, and 48.08%, (engineering) loading strain, respectively.

**Figure 17:**
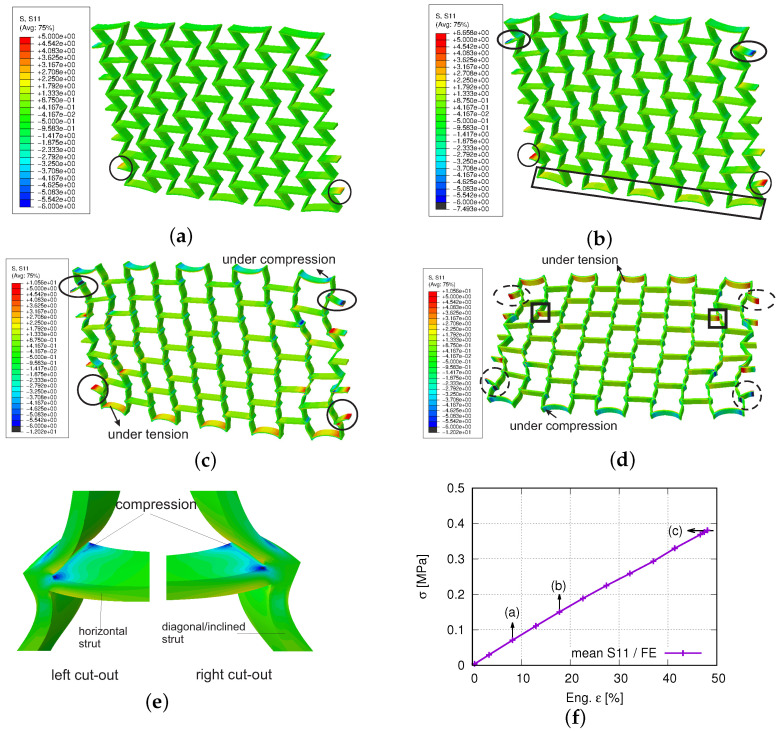
FE -predicted loading direction stress in deformed auxetic structure: (**a**,**b**) at 8.18% and 17.80% (engineering) loading strains, respectively; (**c**,**d**) at 48.08% loading strain in two different perspective views; (**e**) the zoom-in view of the two cut-outs in (**d**), but in another perspective view; (**f**) mean value evolution of loading direction stress v.s. (engineering) loading strain.

**Figure 18:**
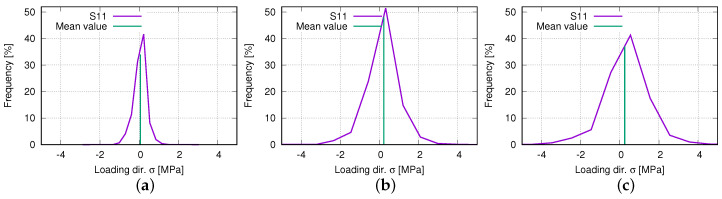
Histogram of FE-predicted loading direction stress of deformed auxetic structure with 5 × 5 cells at loading (engineering) strains: (**a**) 8.18%; (**b**) 27.42%; (**c**) 48.08%.

Figure 16a plots the experimental and FE-predicted force–displacement curves, where the marked three points correspond to the loading status for subfigures Figure 16b–d. Figure 16b–d present the von Mises stress distribution at the deformed status of (engineering) strains 3.37%, 27.42%, and 48.08%. Figure 17a–c demonstrate the loading direction stress distribution at 8.18%, 17.80%, and 48.08% (engineering) loading strain. Figure 17d is reported at the same global loading as Figure 17c, merely in another perspective view to show the coexistance of the tensile and compressive stress in the auxetic structure. Figure 17e shows another perspective view for the two cut-outs marked with squares in Figure 17d. In Figure 17f, the three marked points correspond to deformed states of Figure 17a–c, respectively. The histograms of the loading direction stress distribution are presented in Figure 18a–c at 8.18%, 27.42%, and 48.08% loading (engineering) strains. A more detailed discussion of the results, concerning the auxetic structure deformation behavior, will be given in the following section.

## 5. Discussion

The current study objects are the auxetic structures made of PBAT, a simple case of metamaterials, since no electrically or magnetically effective materials are inserted inside them; also, neither permittivity nor permeability is considered. Metamaterials refer to both materials and structures. In the current case, auxetic structural deformation behavior can also be called metamaterial deformation behavior. In this work, the former is preferred, since the investigation goal is the auxetic structure deformation behavior and topology optimization, whereas the latter will be presented in further work. The current results cover the force–displacement and stress–strain flow curves, the Poisson’s ratio evolution, structural and statistical stress distribution, and auxetic behavior, emphasizing the auxetic deformation characteristics. Figure 13, Figure 14, Figure 15, Figure 16, Figure 17 and Figure 18 show the FE predictions, without considering the residual stress and warpage. These figures also include the measured results.

The FE-predicted displacement-force curve matches the experimental flow behavior well (Figure 13), even though the numerical one behaves softer than the reality at a given loading. During the trial simulations with the selected material model (Ogden model with N = 4), both types of solid and hybrid elements, “C3D8” v.s. “C3D8H” (ABAQUS), are used, since both are applicable. It was found that “C3D8H” leads to numerical predictions nearer to reality. In the present case, the other type causes a much higher strength response than the real one. Figure 13 implies that using the standard geometry (Figure 1) to find out a suitable material model, which will be further used in the simulation for the auxetic behavior prediction, is applicable. In Figure 14, it refers only to the curves calculated from three rows (rows 2–4 in Figure 3) for the well-comparable Poisson’s ratio between the experiment and the simulation. The curve calculated from all five rows proves that the upper and lower rows negatively influence the accuracy of the prediction of the auxetic structural Poisson’s ratio. The reason is that the general auxetic deformation behavior is strongly disturbed in these two rows (row 1 and row 5 in Figure 3) by the BCs. The FE prediction of Poisson’s ratio matches the measured data well (Figure 14). As mentioned in Section 4, the optimum Poisson’s ratio is −1.0 in this work. Poisson’s ratio increases according to the loading for the auxetic structure, and the range is about [−1.0, −0.8]. This means that the expansion ratio of the lateral and loading direction decreases. Still, the ability of the auxetic deformation is good until about 15% tensile loading strain, while a Poisson’s ratio with a value of −0.8 is taken to be near −1.0. Influenced by BCs, it is evident that the cells at the end rows, neighbors of clamping jaws, deform more than cells in the other rows from the experimental results (Figure 15a,b), especially the four cells on the four corners. The free struts of the cells on the outer side generally are arc-formed under loading. This effect is due to that such struts do not have neighbors, which means no constraints from neighbor cells, and more space is available for their deformation. The four struts on four corners of the specimen also rotate much (around the z-axis, coordinate see Figure 15d). In addition to the less constrained condition, the BC is also an essential influencing factor for the rotation. The structural deformation characteristics are well-captured by the FE simulation (Figure 15c,d). The measured stress distribution in the auxetic structure is not available, but it is easily obtained from the numerical prediction (Figure 15c,d). In the whole structure, the joining positions of horizontal and inclined struts present relatively high stress. Here, regions with high stress and with high strain are coincident. One factor causing the high stress should be the complexity of the morphology, which introduces complex deformation constraints for the materials. To maintain compatibility, the materials in these positions must deform more, causing higher strain and stress. From Figure 15e, it is obvious that the mean value of the von Mises stress increases according to the loading. Furthermore, from 41% strain on (end loading 48.1%), the von Mises stress increment ratio reduced (the stress increases further). Before this turning point (41% strain), the stress increases averaged about 0.0625 MPa per 1% loading strain. Near to the end of the loading (46.5% to 48.1% strain), the output points appear much denser in a given loading region, which means more iterations are necessary to solve the whole system matrix. It implies that the deformation is more and more difficult, i.e., the auxetic deformation ability goes to the end. From Figure 15f, the auxetic structure’s deformed status is compared between the FE simulation and the experiment. It shows that the BCs strongly influence the deformation of cells in the first and fifth rows (numbering see Figure 3). This influence negatively affects the total auxetic structural deformation behavior, since the predicted curve of the Poisson’s ratio evolution with consideration of these two rows behaves worse than without, compared to the experimental one. The numerically predicted curves in Figure 14 can prove this conclusion. In row one and five, the four cells at the ends of both rows are the most distorted ones. The outer struts of each row illustrate the highest deformation, since the original linear geometry turn out to be arc-shaped. The stress distribution of the FE simulation (Figure 14) is highly inhomogeneous. The junction places of the inclined struts mostly show higher stresses than other places.

Figure 16a shows the force evolution of the whole auxetic structure according to the applied displacements. The values marked with (b–d) present the force needed to maintain the deformed status given in Figure 16b–d, respectively. As mentioned above, the FE result captures the general characteristics of the measured data, i.e., non-linear increasing (applied displacement increases linearly). The exact reasons, which cause the softer behavior in the simulation than in the experiment, are unclear at the moment. The BCs might be one reason. In reality, the displacement is applied to clamping jaws, and then the clamping jaws drag the bars in row 1 and row 5 and further to other rows. In the FE prediction, the displacement is directly applied to the horizontal struts (bars) in row 1 and 5. This difference might lead to some artificial effects, resulting in a softer predicted behavior. However, in the authors’ opinion, this BC difference between the simulation and the experiment is not the primary reason. Possibly, the reason is the not an accurate enough description of the auxetic structure deformation in the simulation. There are no special functions to describe this auxetic deformation behavior. The numerical prediction is the result of solving the whole system matrix in the FE simulation, which includes the material model, material parameters, BCs, force/stress equilibrium, displacement/strain compatibility conditions, and other ones. However, no explicit functions, which consider the auxetic geometric parameter and depict the cells’ deformation behavior, are involved in the FE simulation. However, much further study is necessary to achieve the above-mentioned functions. At about 3.37% loading strain (Figure 16b), the structure still possesses good further deformation ability, i.e., deforms auxetically. From Figure 16c, cells in rows 1 and 5 have apparent rotation and distortion, which means the auxetic behavior in such rows/cells is not trustable anymore. At this loading stage, rows 2–4 maintain their auxetic characteristics. Simply, the auxetic deformation ability approaches the upper limit. At approximately 48% strain (Figure 16d), the cells in rows 1 and 5 are further distorted, and the diagonal struts in rows 2 and 4, marked with two rectangles, are also distorted. In the authors’ opinion, the auxetic behavior is exhausted. Any further loading is nothing else as merely structural elongation, and the extent of this elongation is material- and layer connectivity-dependent.

Figure 17 illustrates the loading direction stress distribution and its mean value evolution, according to the loading. Figure 17a–d shows that both tensile and compressive stress exist. At 8.2% loading (engineering) strain, the two circles in Figure 17a marked the highest tensile stress position, the opposite side showing compressive stress. This tension–compression coexistence is more evident in Figure 17b–d. Figure 17b is plotted at 17.8% loading strain. The two circles in Figure 17b present the inner side of the strut, while the two ovals are the outer sides, where the former present tension and the latter compression. The stress distribution has a characteristic of symmetry, according to the geometrical middle line parallel to the loading axis. The rectangle in Figure 17b denotes the high tensile region, while the opposite side is under compression. Figure 17c,d are under the same load (48.08% strain) with different perspective views. The two solid circles (Figure 17c) present tensile stress, and the two dashed (Figure 17d) present compressive stress. The ovals in Figure 17c,d denote a similar behavior as the circles. By comparing the stress distribution marked in circles and ovals (Figure 17c,d), the symmetric stress distribution is apparent. Generally, one side of the inclined struts shows tension, while the opposite side compression. This tension–compression coexistence can be seen by comparing the square cut-outs in Figure 17d,e. Figure 17e illustrates the other side of the same horizontal strut as marked in the squares in Figure 17d, but with a zoomed-in view. According to the (engineering) loading strain, Figure 17f plots the mean value evolution of the stress in the loading direction by considering the element volume weighting factor. The mean value reaches 0.38 MPa. In the deformed structure, both the tensile and compressive stress exist locally (Figure 17). During the mean value calculation, the positive and negative values compensate for each other, which causes the low mean value.

Figure 18a,c denotes the histogram of the loading direction stress (S11) at 8.18%, 27.41%, and 48.08% (engineering) loading strains, respectively. At low-loading strains (Figure 18a), the stresses concentrate in a small region, and the mean value is very near to zero MPa. The stress range enlarges according to the increased loading, which can be seen by comparing the widths of the curves in Figure 18a–c, but the mean value does not increase much. The slight mean value increment is caused by the nearly symmetric distribution of the histograms, which means the tensile and compressive stresses in the structure increase simultaneously and nearly symmetrically. As mentioned above, the mean value is compensated by the positive and negative stresses. The highest frequency also increases from 8.2% (Figure 18a) loading strain to 27.4% (Figure 18b), but decreases from 27.4% to 48.8% (Figure 18c). In the loading ranges with both the frequency increment and decrement, the stress inhomogeneity and (absolute) maximum stress increase. In the loading ranges with the frequency increment (Figure 18a,b), still, a large number of elements possess stresses near the mean value. While in the loading ranges with the frequency decrement (Figure 18b,c), more and more elements show a more significant discrepancy of the stress value to the mean value, which is accompanied by the exhausted auxetic deformation behavior.

In addition to plastics, the base material for the auxetic structure can be metallic. Generally, auxetic structures made of metals possess better resistance to temperature, higher strength, and more stable properties in the long run. Still, the additive manufacturing (AM) methods are particularly material-dependent (a non-AM method is also possible). For structures made of metallic materials, one consideration point influencing the structural deformation behavior is porosity. Box et al. [56] reported an auxetic metamaterial fabricated from hard material by perforating metals (or plastic sheets). Based on their experimental and numerical results, it concluded that the behavior of hard structures is dominated by elastic deformations of the structural elements comprising the microarchitecture (the local holy architecture composing the whole auxetic structure). Xue et al. [57] produced their auxetic structures made of Al and its alloy by 3D printing combined with the molten metal infiltration technique. Under the compressive mechanical loading until about 50% strain, the stress–strain flow curves of (auxetic) specimens made of pure Al and 6063 Al alloy also show three regions, a liner elastic, a plateau, and a slightly increasing and densification region. This three-zone characteristic shares the same tendency as the current study (Figure 5a) auxetic structure made of PBAT under tension. Ulbin et al. [58] numerically studied the fatigue behavior of auxetic cellular structures made of AlSi10Mag alloy using selective laser melting (SLM). One remarkable finding is that less auxetic structure (higher Poisson’s ratio) tends to possess a better fatigue life expectancy. Meena and Singamneni [59] compared the auxetic deformation behavior of specimens with standard re-entrant and S-shaped structures produced by selective laser melting 316 L stainless steel powders. They found that the latter has reduced stress concentration effects and exhibits better auxetic response. This work [59] highlighted that the stress distribution pattern and concentration (results of this study) fill the gap of auxetic structure/behavior investigation, since most works concentrate on the auxetic response. The current work (Figure 15, Figure 16, Figure 17 and Figure 18) discussed stress concentration and distribution based on structure and statistics. Mauko et al. [60] studied the dynamic deformation behavior of chiral auxetic lattices at low and high strain rates, where the specimens were produced with the powder bed fusion method from austenitic stainless steel (SS 316L-0407).

In the near future, the auxetic structure deformation will be simulated considering residual stress caused by the cooling down process. The optimization of the auxetic topology (a unit cell) using the FE method will be presented. One of the next investigations will be searching for suitable material models from ABAQUS inherent models for the PLA and PLA-PBAT blends. With tomographic images scanned at different loading steps, one emphasis will be to identify the impurities and their evolution inside the auxetic structure using machine learning methods. Another machine learning result serves as the intermediate status of the auxetic structure between two scans. Such machine learning results can be compared with the simulated ones at the same loading level.

## 6. Conclusions

The current work studies the auxetic structural (metamaterial) deformation behavior of PBAT biodegradable polymers. Three different methods for Poisson’s ratio calculation are applied by using the measured data, where the suitable one is also used in handling FE results. The selection of a suitable ABAQUS inherent material model is the starting point, for which the tensile stress–strain curve is used by comparing the FE predicted and experimental results. Then, the auxetic tensile deformation behavior is studied by using FE simulation, the results of which are compared with those of experiments. It leads to the following conclusions: the material model Ogden (N = 4, ABAQUS) is found to be the most suitable one to simulate the PBAT tensile deformation behavior. The result of this work suggests that the top and bottom rows (neighboring two rows of clamping jaws) should not be considered for the Poisson’s ratio calculation of the whole structure, due to the BCs’ influence. An auxetic structure with 5 × 5 cells is the optimal one. Concerning the auxetic behavior, the highly non-linear behavior of the force–displacement curve obtained from the experiment is well-captured by the FE prediction, where the numerical result behaves a little softer than the reality. The Poisson’s ratio evolution predicted by FE simulation matches the experimental one very well. By overlapping the deformed status of the auxetic structure from the test and the FE calculation, it shows that the real auxetic deformation characteristics can be predicted in morphological detail using FE simulations. The numerical prediction shows that the whole auxetic structure deforms symmetrically according to the structure’s middle line parallel to the loading axis. Regions under tension and compression coexist in a single strut. Due to this coexistence, the statistical mean values in the histograms increase very slightly, since the tensile and compressive stresses compensate for each other.

## Figures and Tables

**Table 1:** Injection molding parameters used for the tensile specimen production.

Content	Speed	Speed	Distance				Time				
Symbol	n	v${}_{s}$	s${}_{U}$	s${}_{D}$	s${}_{M}$	s${}_{P}$	t${}_{Z}$	t${}_{E}$	t${}_{N}$	t${}_{K}$	t${}_{D}$
Unit	m/min.	cm${}^{3}$	cm${}^{3}$				s				
PBAT	7	30	14.8	45	11.2	4	67.61	1.15	30	30	9.75
**Content**	**Pressure**			**Temperature**							
Symbol	p${}_{E}$	p${}_{N}$	p${}_{ST}$	t${}_{Z1}$	t${}_{Z2}$	t${}_{Z3}$	t${}_{Z4}$	t${}_{Z5}$	t${}_{n}$	t${}_{W1}$	t${}_{W2}$
Unit	bar			${}^{\circ }$C							
PBAT	824	500	60	50	160	180	185	190	200	40	40
n: rotatinal speed of filament screw						v${}_{s}$: injection speed					
s${}_{U}$: changeover point						s${}_{D}$: metering stroke					
s${}_{M}$: mass cushion						s${}_{P}$: decompression					
t${}_{Z}$: cycle time						t${}_{E}$: injection time					
t${}_{N}$: holding pressure time						t${}_{K}$: cooling time					
t${}_{D}$: dosing time						p${}_{E}$: injection pressure					
p${}_{N}$: holding pressure						p${}_{ST}$: back pressure					
t${}_{Z1}$–t${}_{Z5}$: cylinder temperature 1–5						t${}_{n}$: nozzle tempertature					
t${}_{W1}$: mold temperature gate side						t${}_{W2}$: mold temperature clamping side					

**Table 2:** Printer model and parameters used for the auxetic specimen production.

Printer model:	Prusa i3 MK2
Nozzle diameter:	0.4 [mm]
Extrusion width:	0.44 [mm]
Printing temperature:	200 [${}^{\circ }$C]
Plate temperature:	60 [${}^{\circ }$C]
Layer height:	0.2 [mm]
Print speed:	35 [mm/s]
infill pattern:	triangles (25% infill only in clamping bars)

**Table 3:** Parameters of Ogden Model (N = 4) evaluated by ABAQUS-CAE under constant temperature by using options of unixial test data and isotropic material.

Parameter	Value	Parameter	Value	Parameter	Value
N	4	ν	0.41	-	-
$\alpha_{1}$:	5.2798	$\mu_{1}$	−32.7303	$D_{1}$	0.01112
$\alpha_{2}$:	5.2803	$\mu_{2}$	1.7398	$D_{2}$	0.0
$\alpha_{3}$:	−1.0637	$\mu_{3}$	3.5032	$D_{3}$	0.0
$\alpha_{4}$:	−10.5607	$\mu_{4}$	61.9267	$D_{4}$	0.0

## Data Availability

Data are contained within the manuscript.
